# Engineering *Thermotoga maritima* β-glucosidase for improved alkyl glycosides synthesis by site-directed mutagenesis

**DOI:** 10.1093/jimb/kuab031

**Published:** 2021-06-14

**Authors:** Yemin Xue, Mengke Xue, Fang Xie, Mengchen Zhang, Hongyang Zhao, Tao Zhou

**Affiliations:** Department of Food Science and Engineering, College of Food and Pharmaceutical Engineering, Nanjing Normal University, Nanjing 210023, P. R. China; Department of Food Science and Engineering, College of Food and Pharmaceutical Engineering, Nanjing Normal University, Nanjing 210023, P. R. China; Department of Food Science and Engineering, College of Food and Pharmaceutical Engineering, Nanjing Normal University, Nanjing 210023, P. R. China; Department of Food Science and Engineering, College of Food and Pharmaceutical Engineering, Nanjing Normal University, Nanjing 210023, P. R. China; Department of Food Science and Engineering, College of Food and Pharmaceutical Engineering, Nanjing Normal University, Nanjing 210023, P. R. China; Department of Food Science and Engineering, College of Food and Pharmaceutical Engineering, Nanjing Normal University, Nanjing 210023, P. R. China

**Keywords:** β-Glucosidase, Alkyl-glucosides, *Thermotoga maritima*, Transglycosylation mutants

## Abstract

Alkyl glycosides are well-characterized nonionic surfactants, and can be prepared by transglycosylation reactions with retaining GH1 glycosidases being normally used for this purpose. The produced alkyl glycosides can also be hydrolyzed by the glycosidase, and hence, the yields of alkyl glycosides can be too low for industrial use. To improve the transglycosylation-to-hydrolysis ratio for a β-glucosidase from *Thermotoga maritima* (TmBglA) for the synthesis of alkyl glycoside, six mutants (N222F, N223C, N223Q, G224A, Y295F, and F414S) were produced. N222F, N223C, N223Q, G224A improved catalytic activity, F295Y and F414S are hydrolytically crippled with *p*-nitrophenol-β-d-glucopyranoside (*p*NPG) as substrate with an 85 and 70-fold decrease in apparent *k*_cat_, respectively; N222F shows the highest *k*_cat_/*k*_m_ value for *p*NPG. The substrate selectivity altered from *p*NPG to *p*NP-β-d-fucoside for N222F, F295Y, and F414S and from cellubiose to gentiobiose for N222F and F414S. Using *p*NPG (34 mM) and hexanol 80% (vol/vol), N222F, Y295F, and F414S synthesized hexyl-β-glycoside (HG) yields of 84.7%, 50.9%, and 54.1%, respectively, HG increased from 14.49 (TmBglA) to 22.8 mM (N222F) at 2 hr by 57.42%. However, this higher transglycosylation effect depended on that three mutants creates an environment more suited for hexanol in the active site pocket, and consequently suppressed its HG hydrolysis.

## Introduction

Alkyl glycosides are well-characterized nonionic surfactants and have already been widely used in food, pharmaceutical and cosmetic and detergent industries due to their unique features such as high surfactant performance, low toxicity, antimicrobial activity, and good biodegradability (Anonymous, 2012; Liu et al., 2013; Matsumura et al., 1990; Noritomi et al., 2013; Rybinski & Hill, 1998). These compounds can be prepared by both chemical and enzymatic synthesis (Mohd & Saroj, 2013; Papanikolaou, 2001). However, chemical synthesis of alkyl glycosides involves extremes of temperature, pressure, use of toxic catalysts, and multistep reactions as well as anhydrous conditions. Therefore, enzymatic synthesis has attracted more attention in recent years (Guo et al., 2015; Turner et al., 2007). Lately, β-glucosidases have received great interest because of their biosynthetic abilities and their various biological functions, including improving bioavailability (converting bioactive glycosides into their corresponding aglycones) (Sun et al., 2014; Xue et al., 2009), food detoxification (Chen et al., 2014), flavor enhancement in wines (Gueguen et al., 1996), cellulose converting to bioethanol (Abedinifar et al., 2009), synthesizing chemical compounds (Guo et al., 2015; Hansson & Adlercreutz, 2001; Mladenoska et al., 2007; Park et al., 2005). As compared to glycosyl transferases, glucosidases may exhibit broad specificity with respect to their natural substrates or engineered substrates that broaden their applications and exhibit wide substrate specificity and high stereo-selectivity to be obtained without any tedious protection or expensive nucleotide-activated sugars as glycosyl donors (Guo et al., 2015; Hansson & Adlercreutz, 2001; Mladenoska et al., 2007; Park et al., 2005).

**Fig. 1:**
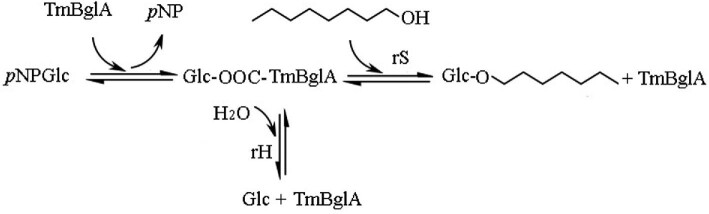
Reaction scheme for the conversion of *p*-nitrophenol-glycoside to hexyl-β-glycoside in hexanol (transgalactosylation reaction) by *T. maritima* β-glucosidase TmBglA. The synthesized hexyl-β-glycoside can be hydrolyzed by the enzyme (secondary hydrolysis). The conversion to glucose with water as the nucleophile (hydrolysis reaction) was a side reaction.

Family 1 glycosyl hydrolase (GH1), which can catalyze the transglycosylation reaction as well as hydrolysis, is ideal enzyme for use in the synthesis of glycoside derivatives, their proportion of transglycosylation to hydrolysis varies depending on the different sources of enzymes (Guo et al., 2015; Hansson & Adlercreutz, 2001; Park et al., 2005). The proposed reaction scheme for transglycosylation using *p*-nitrophenol-glucoside as the glycosyl donor and hexanol as the acceptor is depicted in Fig. 1, where the β-glucosidase cleaves the *p*-nitrophenol-glucoside and forms glucosyl–enzyme complex with the liberation of *p*-nitrophenol. The free enzyme is regenerated by transferring the glucosyl moiety to water (hydrolysis for forming glucose) or to hexanol [transglycosylation for forming hexyl-β-glycoside (HG)]. In most cases, yields of alkyl glycosides are very low; presumably the transglycosylation products are substrates for the enzyme and undergo hydrolysis. To obtain high synthesis yield, glycosidase with high ratios of transferase to hydrolase activity was needed. Pioneering studies have revealed the innate property and molecular configuration in the active sites of glucosidase creates an environment with hexanol outcompeting water as a glycosyl acceptor, and consequently suppresses their hydrolysis of transglycosylation products; thus favors transglycosylation reaction (Frutuoso & Marana, 2013; Hansson & Adlercreutz, 2001; Lundemo et al., 2013, 2017; Mladenoska et al., 2007). Thus, directed evolution could be a more effective approach to enhance transglycosylation and suppress hydrolysis; and the efforts to engineer glucosidase for improved transglycosylation have been reported. Lundemo et al. (2013) enhanced transglycosylation activity of a glycoside hydrolase family 1 from *Thermotoga neapolitana* by introducing a mutation of N220 to phenylalanine in the aglycone subsite; Yang et al. (2017) enhanced synthesis of galactooligosaccharides by a mutation in hyperthermophilic *Thermotoga naphthophila* RKU-10 β-glucosidase, F414S, resulting the hydrolytic activity deduction by up to 350-fold; Feng et al. (2005) reported that mutations F401S and N282T increased the yield of trisaccharides from *Thermus thermophiles* β-glycosidase by 6-fold compared with the wild-type enzyme. Hansson & Adlercreutz (2001) showed that mutations M424K and F426Y significantly improved (18–40%) the synthesis of galactooligosaccharides from the *Pyrococcus furiosus* β-glucosidase. Berrin et al. (2003) reported a mutation in human cytosolic β-glucosidase, F225S, resulting a complete removal of all hydrolytic activities. Choi et al. (2008) reported three mutants in a *T*. *neapolitana* β-glucosidase, N291T and N291T/F412S, produced transglycosylation/hydrolysis ratios about 3- and 8-fold higher, respectively, than that of the wild-type enzyme. Higher ratios of transglycosylation have been seen in the above mutational studies in the aglycone (+1) and glycone (−1) subsites of β-glucosidases (Hansson & Adlercreutz, 2001; Hassan et al., 2016; Lundemo et al., 2017; Teze et al., 2014; Tran et al., 2010). Therefore, engineering the protein structure is an efficient approach to improve the yield of the transglycosylation products of glycosidases.

For engineering, it is advantageous to start with the hyperthermophilic enzyme that has highly stable and robust enzymes for bioprocesses that are relevant for the desired application (Lundemo et al., 2013, 2017; Vieille & Zeikus, 2001). Consequently, a β-glucosidase from *Thermotoga maritima*, TmBglA, which composed of retaining glycosidases with broad substrate specificity, and exhibit a relatively broad acceptor-substrate specificity with simple substrates, is an ideal starting point for protein engineering studies. By using results from our previous work and other mutational studies of β-glycosidases along with structural information from a recently generated mode, in this investigation, we have generated six TmBglA active-site variants and present their biochemical characterization aimed at improving alkyl glycosides production. Several mutants were observed to improve both transglycosylation activity and stability, as well as ethanol tolerant. Molecular docking between mutants and substrates or products for an energetically favorable conformation for the complex were obtained using computer simulation and compared with that of wild-type β-glucosidase to investigate the structural changes responsible for the improved characteristics.

## Materials and Methods

### Materials and Chemicals

High-performance liquid chromatography (HPLC) grade acetonitrile and methanol were purchased from Fisher Scientific (Hanover Park, IL). *p*-Nitrophenol (*p*NP) and *p*-nitrophenol glycoside substrates were purchased from Sigma-Aldrich (St Louis, Missouri, USA). Cellobiose, lactose, gentiobiose, and HG were purchased from Sangon Biotech and Aladdin Chemical Co (China).

### Cloning and Site-Directed Mutagenesis

Plasmid constructions were carried out according to standard procedures (Sambrook et al., 1989). The *T. maritima* β-glucosidase A gene, *bglA*, based upon that reported in Genbank entry X74163, was amplified with pET-20b*-bglA* (Xue et al., 2009). Mutagenesis was performed in order to introduce the following designed changes N222F, N223C, N223Q, G224A, Y295F, and F414S, respectively. Six oligonucleotides for each mutation were designed to contain the corresponding nucleotide changes (see **Table** **1**). These oligonucleotides and pET20b-*TmbglA* as template were used to introduce mutations using PCR under the following conditions: one cycle of denaturation at 95°C for 5 min, 30 cycles of denaturation at 94°C for 40 s, annealing at 55°C for 40 s, extension at 72°C for 4 min, and extra extension at 72°C for 7 min. The PCR products were purified using the QIAquick PCR purification kit, and phosphate and ligated with T4 polynucleotide kinase resulting in the expression vectors containing exchange mutant TmBglA. The nucleotide changes were then sequenced by Biological Services Unit of Shang Hai.

**Table 1.** Mutagenic Primer Sequences

Primer	Mutation	Direction	Sequence(5′–3′)
1	N222F	Forward	AGTTTTC**TTC**AATGGATATTTCGAACCTGC
		Reverse	ATTCCGATCTTTCCATCTTTCACG
2	N223C	Forward	AGTTTTCAAT**TGC**GGATATTTCGAACCTGCG
		Reverse	ATTCCGATCTTTCCATCTTTCACG
3	N223Q	Forward	AGTTTTCAAT**CAG**GGATATTTCGAACCTGCG
		Reverse	ATTCCGATCTTTCCATCTTTCACG
4	G224A	Forward	AGTATTCAACAAC**GCG**TATTTCGAACCTGCGAGT
		Reverse	ATTCCGATCTTTCCATCTTTCACG
5	Y295F	Forward	AACTAT**TTT**TCCGGTCATTTGGTGAAG
		Reverse	CAATCCAACAAAGTCGATCTTTTC
6	F414S	Forward	AGA**TCT**GGTATTGTGTATGTAGACTAC
		Reverse	CTTGGAATATCCCTCTGCCCATTC

*Note.* Bold residues indicate the sites of mutation.

### Expression and Purification of β-Glucosidase

*E. coli* BL21(DE3) harboring various expression plasmids containing pET20b-*bglA* and mutational plasmid were cultured for production of TmBglA and mutants, all variant enzymes were purified to homogeneity as judged by SDS-PAGE analysis under the same conditions as previously described (Sun et al., 2014; Xue et al., 2009).

### Hydrolysis Activity Assay

Protein concentration was determined by the Bradford method (Bradford, 1976), using bovine serum albumin (BSA, Sigma) as a standard. Enzyme activity was quantified by *p*-nitrophenol (*p*NP) release from *p*NPG, *p*NPGal, and *p*NPFuc (Sigma, USA). To 190 μl of 2 mM *p*NPG, *p*NPGal, and *p*NPFuc in 50 mM potassium phthalate buffer (PPB, pH 6.2), 10 µl of suitably diluted enzyme was added and incubated for 5 min at 90°C. After the completed incubation, the reaction was stopped by the addition of 600 µl of 1 M Na_2_CO_3_, and the amount of *p*NP released was measured at 410 nm against a blank. One unit was determined as the amount of enzyme producing 1 μmol of *p*NP per minute under the above assay conditions. For oligosaccharides, 0.5 µg of the enzyme was added in 50 µl of the total reaction volume. The reaction was stopped by heating the sample to 100°C for 10 min. The amount of glucose liberated was measured using a Glucose Assay Kit (Nanjing Jiancheng Bioengineering Institute) in accordance with the manufacturer's protocol. All reactions were done in triplicate, and the relative standard deviation was <2%.

### Biochemical Characterization

To determine the effect of pH and temperature on the enzyme activity, the enzyme was incubated in the pH range of 4.2–8.2 at 90°C, and temperatures were varied from 35°C to 100°C for pH 6.2, and then the residual activities were evaluated. Buffers used: pH 4.2–6.2:50 mM citrate buffer; pH 5.8–8.2:50 mM phosphate buffer; pH8.2–9.0:50 mM Tris–HCl buffer. Thermostability was determined at temperatures range of 50–95°C in 50 mM phosphate buffer (pH 6.2). The effect of pH on enzyme stability was monitored by placing the enzyme solution at pH range of 4.2–8.2 at 37°C for 1 hr. The effect of ethanol on the enzyme activity was evaluated by determining the relative activity of the wild-type TmBglA and mutants towards *p*NPG in different concentrations of ethanol (5–30%, vol/vol) at 37°C for 1 hr. Full activity was determined at each pH value. The activity of the enzyme without preincubation was defined as 100%. Activity was determined at their optimal pH and temperature. Data are expressed as the means of three experiments and the standard deviation for the mean was recorded to be <2%.

For determinations of the kinetic parameters (*K*_m_, *V*_max_, *k*_cat_/*K*_m_) of *p*NPG, reaction was carried out at the optimum conditions using 0.2–2.0 mM *p*NPG. Kinetic parameters, *K*_m_ and *V*_max_, were determined by the Lineweaver–Burk representation of the Michaelis–Menten model. The catalytic efficiency, *k*_cat_/*K*_m_, was calculated to determine the substrate specificity of each enzyme. To calculate the catalytic constant, apparent *k*_cat_, the subunit molecular mass of 52,338 Da for TmBglA and mutants were used. Each experiment was done in duplicate, and measurements were made in triplicate. The standard error was recorded to be <2 percent.

### Transglycosylation Reaction

To evaluate the transglycosylation activities, 2.0 μg of purified TmBglA and mutants were added to the 0.2 ml of reaction mixtures containing 34 mM *p*NPG, 80% (vol/vol) hexanol and 50 mM sodium phosphate buffer (pH 6.2), respectively, and allowed to react at 60°C in a Thermo Mixer apparatus (WHY-2 Jiangsu Jinchengguosheng instrument Co. China) with shaking at 150 rpm. The reaction mixtures were left for 20, 40, 60, 120, 240, and 480 min, and then were stopped by cooled in ice bar before analysis and centrifuged at 8,000 rpm and 4°C for 5 min to get the supernatant, and diluted with 200 μl acetonitrile and 20 μL of each sample was injected for high-pressure liquid chromatography (HPLC) to monitor the enzymatic synthesis process. Each assay was done in duplicate, and the standard deviation was <5% from the mean.

### Analytical Methods

For TLC analysis, the detection and identification of hydrolysis and transglycosylation products were carried out by TLC analysis. Three-microliter aliquots of the reaction mixture were spotted on thin layer chromatography plates using silica gel 60 F_254_ (Yantai Jiangyou Silica Gel Development Co., Ltd. China), and developed with acetonitrile/water (8/2 vol/vol), the different spots were visualized under UV light at 254 nm. The sugars on the plate were stained by spraying the air-dried plate with a solution containing H_2_SO_4_/methanol (1:9), and heating it at 100°C for 10 min.

HPLC analysis of the reaction products, substrates and products of synthesis were analyzed in an Agilent1100 ZORBAX SB-C18 column (4.6 × 150 mm, 5 μm) HPLC delivery system, provided with refractive index (RI) detector under the following conditions: column temperature: 30°C, mobile phase: 60:40 acetonitrile/water, flow rate: 1.0 ml/min. Both HG and *p*NPG elute after 1.6 min and was measured by a RI detector, and *p*NP has a retention time of 2.2 min. Meanwhile, *p*NPG elutes after 1.3 min and was followed at 405 nm as well as with the UV detector, and *p*NP have a retention time of 2.0 min but HG does not absorb at 405 nm. Because of the coelution of one substrate *p*NPG and the reaction product HG, the product formation HG was estimated according to the following means: The peak area of *p*NPG in UV detector correlate with those in RI detector was determined, and their peak area in RI detector are calculated, and was subtracted from the total peak area of *p*NPG/HG (ret. time: 1.6 min) in RI detector, and the residual peak area was used as the peak area of HG in RI detector to estimate the product HG formation in the sample.

### LC–MS Analyses

LC–MS analyses of the reaction product were carried out by using a system consisting of 6460 QQQ MS (Agilent, USA) and 1290 Infinity LC (Agilent, USA). Samples were filtered (0.45 μm, Millipore) and 10 μl was directly injected. The separation was performed using a ZORBOX-C18 reverse phase column (4.6 × 250 mm; 5 μm; Agilent, USA) protected by a pre-column with acetonitrile-water pre-mixed (60:40, vol/vol) solvent as the mobile phase at a flow rate of 0.6 ml/min. Mass spectrometry was performed in ESI source negative mode (50–400). The ion source temperature was set at 300°C. The capillary voltage and fragmentor were set at 3.5 kV and 135 V, respectively. Nebulizer gas was set at 45 psi. Data acquisition is carried out by Mass hunter.

### Molecular Docking Studies

The molecular structure of substrates *p*NP-β-d-glucoside and hexyl-glucose was constructed using ChemBio 3D Ultral 3.0. AutoDock version 4.2 (http://autodock.scripps.edu) was used for docking simulations. The structure of the protein–ligand docked complex was visualized and analysed using a PyMOL visualization tool. The substrate orientation giving the lowest interaction energy was chosen for docking. The figure was drawn with Accelrys DS Visualizer 3.0.

## Results and Discussion

### Determination of Site Location of TmBglA for Mutagenesis

β-Glucosidase A from *T*. *maritima*, TmBglA, is active upon a broad range of substrates. Similar to other β-glucosidases of GH1, TmBglA share the same tertiary structure, a (β/α)_8_ barrel fold characteristic of GH1. Two highly conserved peptide motifs Thr-Leu-Asn-Glu-Pro (residues 163–167) and Ile-Thr-Glu-Asn-Gly (residues 349–353) are situated opposite to each other inside the active site, more precisely at the ends of strands β4 and β7 containing the typical catalytic residues (E166, E351) (Sun et al., 2014; Xue et al., 2009). As well known, There are differences in the transglycosylation reaction as well as hydrolysis catalyzed by GH1 β-glucosidase, reflecting that the amino acid residues in the glycone (−1) and aglycone (+1) subsides of TmBglA pocket varied with the enzymes. This enzyme TmBglA was chosen for this work as an interesting candidate based on previous promising results that the N222, N223 and G224 at the end of β-strand 5 close to sugar binding sub-site +1 play an important role in determining the substrate specificity of TmBglA (Sun et al., 2014). A close look at the docking results of TmBglA with *p*NGP shows that W168, N222, N246, Y295, and F414 are located around of the glycone (−1) subsites, these amino acid residues were replaced amino acid side chains to improve transglycosylation for related GH1 β-glucosidases in positions previously shown with the aim to enhance the transglucosylation activity and alkyl glycosides yield for TmBglA.

**Fig. 2:**
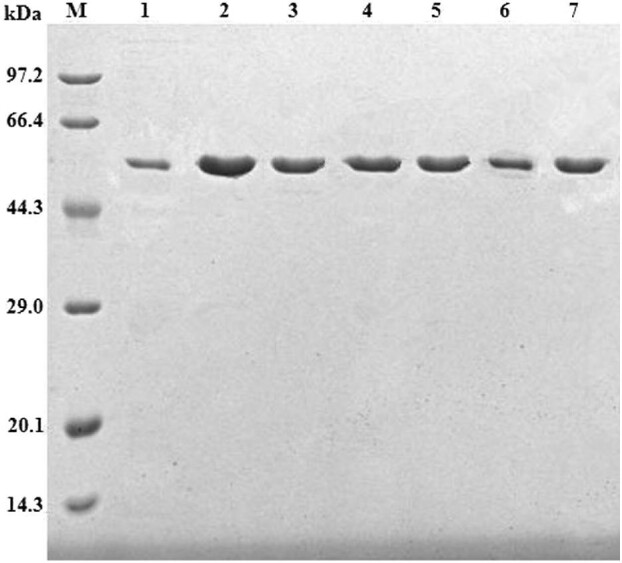
SDS-PAGE analysis of purified TmBglA and mutants. Lane M, molecular mass standards; lane 1, TmBglA; lane 2, N222F; lane 3, N223C; lane 4, N223Q; lane 5, G224A; lane 6, Y295F; lane 7, F414S.

Six expression vectors containing exchange mutant TmBglA were constructed as described in Materials and Methods. The nucleotide sequence of each mutant TmBglA was confirmed by DNA sequencing. These mutant and wild-type TmBglA (WT) were expressed in *E. coli*, purified by immobilized Ni-affinity chromatography after heat treatment and revealed by single bands on SDS-PAGE with apparent molecular masses of 52 kDa (Fig. 2), which were in accordance with the theoretically calculated molecular mass.

### Substrate Specificity of Mutant Enzymes

In order to characterize reaction properties of the mutants, the substrate specificity was measured at 90°C with various aryl-glycosides and disaccharides. Y295F and F414S show significantly impaired catalytic activity toward naturally occurring disaccharides, cellobiose, and lactose as well as aryl-glucoside *p*NPG. N222F, N223C, G224A improved catalytic activity on artificial substrates but not cellobiose and lactose as substrates, whereas G224A improved catalytic activity on artificial and natural substrates except gentiobiose. Two enzymes were also active on positional isomers of cellobiose: gentiobiose (β1→6) (Table **2**). The specific activity of the TmBglA, N223Q, N223C, G224A followed the order *p*NPG >* p*NPFuc > *p*NPGal > cellobiose > gentiobiose > lactose; those of the N222F and F414S followed the order *p*NPFuc > *p*NPGlc > *p*NPGal > gentiobiose > cellobiose > or = lactose; those of the Y295F followed the order *p*NPFuc > *p*NPGlc > *p*NPGal > cellobiose > gentiobiose > lactose; Among all tested substrates, the substrate selectivity for N222F and F414S varied from cellubiose (β-1,4-glucose) to gentiobiose (β-1,6-glucose), and N222F, Y295F, and F414S had a change in substrate selectivity from *p*NPG to *p*NP-β-d-fucoside. The hydrolytic activity of TmBglA and mutants further biochemically characterized as described below.

**Table 2.** The Substrate Specificity of the Wild-Type and Mutant Enzymes

Substrate		Artificial substrate (2 mM)			Natural substrate (2 mM)		
		*p*NPGlc	*p*NPGal	*p*NPFuc	Cellubiose	Gentiobiose	Lactose
Linkage of glycosyl group		β-Glucose	β-Galactose	β-Fucose	β(1,4)-Glucose	β(1,6)-Glucose	β(1,4)-Galactose
Specific activity (U/mg)	TmBglA	731.1 ± 6.82	304.8 ± 9.83	528.4 ± 7.45	26.6 ± 1.09	15.7 ± 0.15	6.8 ± 0.31
	N222F	846.3 ± 8.91	520.7 ± 8.45	954.3 ± 11.78	9.5 ± 0.26	33.4 ± 1.57	0.9 ± 0.08
	N223C	899.7 ± 9.34	331.0 ± 8.37	826.2 ± 9.49	24.7 ± 1.78	9.1 ± 0.16	3.9 ± 0.32
	N223Q	881.4 ± 6.57	266.3 ± 6.59	844.0 ± 9.31	23.5 ± 0.74	9.7 ± 0.31	5.3 ± 0.05
	G224A	750.7 ± 9.93	317.3 ± 3.77	559.7 ± 6.25	42.0 ± 1.78	12.1 ± 0.20	8.9 ± 0.13
	Y295F	4.7 ± 0.16	4.3 ± 0.22	5.1 ± 0.27	0.4 ± 0.01	0.2 ± 0.01	0.1 ± 0.01
	F414S	3.4 ± 0.14	1.3 ± 0.10	5.3 ± 0.26	0.1 ± 0.01	0.3 ± 0.01	0.1 ± 0.01
Relative activity (%)	TmBglA	100	41.7 ± 2.28	72.3 ± 3.64	3.6 ± 0.18	2.1 ± 0.04	0.9 ± 0.03
	N222F	100	61.5 ± 2.32	112.8 ± 7.90	1.1 ± 0.01	3.9 ± 0.12	0.1 ± 0.00
	N223C	100	36.8 ± 1.55	91.8 ± 2.47	2.7 ± 0.09	1.0 ± 0.03	0.4 ± 0.00
	N223Q	100	30.2 ± 2.30	95.8 ± 5.17	2.7 ± 0.07	1.1 ± 0.00	0.6 ± 0.02
	G224A	100	42.3 ± 3.10	74.6 ± 4.78	5.6 ± 0.24	1.6 ± 0.08	1.2 ± 0.05
	Y295F	100	91.3 ± 5.34	108.4 ± 8.71	8.5 ± 0.29	4.2 ± 0.18	2.1 ± 0.09
	F414S	100	38.0 ± 0.69	158.8 ± 7.18	2.9 ± 0.08	8.8 ± 0.32	2.9 ± 0.10

*Note.* Values represent the means ± standard deviation; *n* = 3 (*P* < 0.05).

### Characterization of the Hydrolytic Activity of Mutant Enzymes

**Fig. 3:**
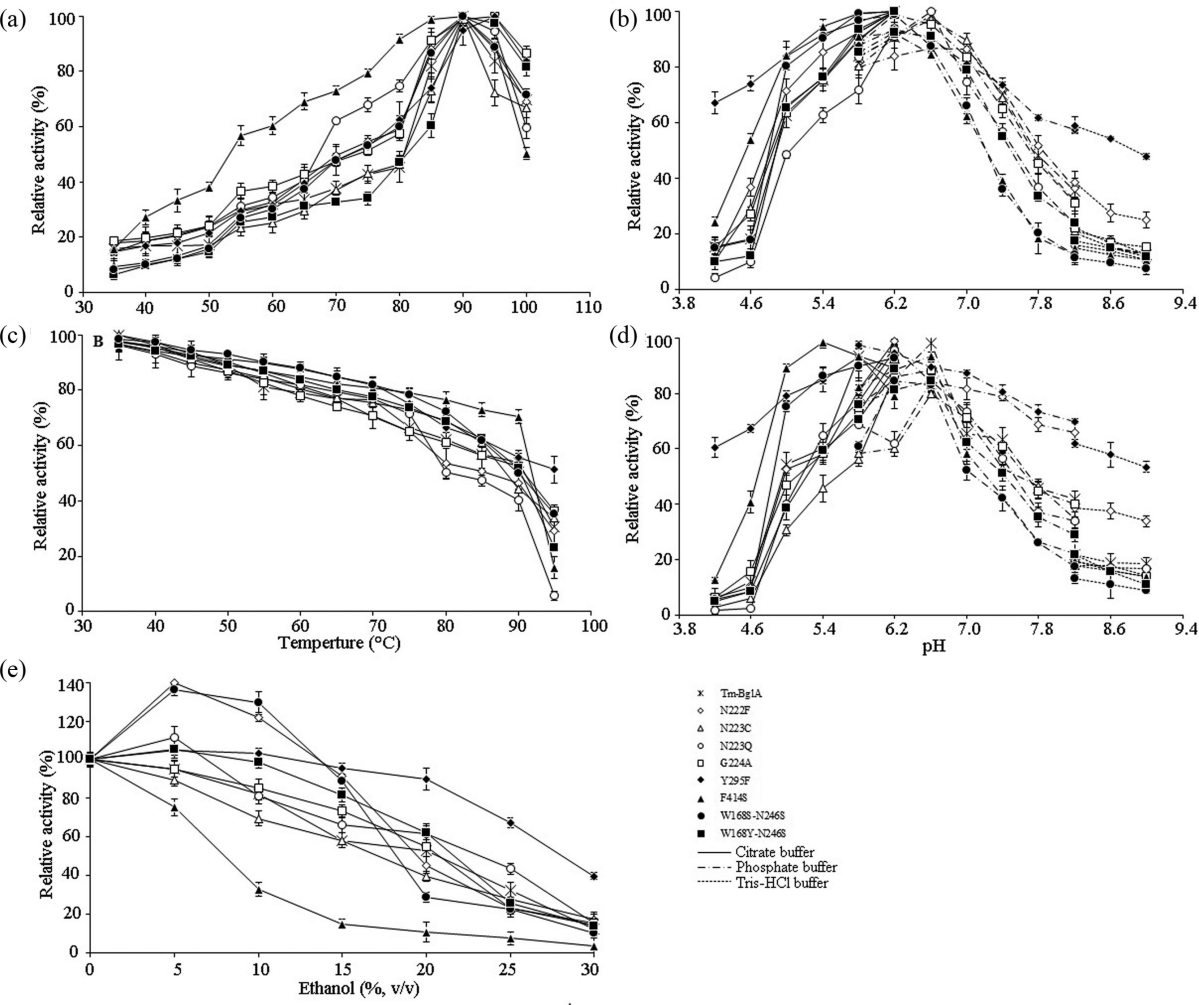
The optimal temperature (a), pH (b), heat stability (c), pH stability (d), and ethanol tolerant (e) profiles of mutant and wild-type β-glucosidases. Temperature dependence at pH 6.2 (a), and pH dependence at 90°C (b) when a 5-min assay was used, the highest level of activity was defined as 100%. For heat stability (c), appropriate volumes of the purified enzyme in 50 mM phosphate buffer (pH 6.2) were perincubated for 1 hr at temperatures range of 50–95°C in the absence of substrate. For pH stability (d), appropriate volumes of the purified enzyme in different buffer (pH 4.2 to pH 8.2) were preincubated for 1 hr at 37°C in the absence of substrate. Buffers used: pH 4.2–6.2:50 mM citrate buffer; pH 5.8–8.2:50 mM phosphate buffer; pH 8.2–9.0:50 mM Tris–HCl buffer. For ethanol tolerant (e), appropriate volumes of the purified enzyme in different concentrations of ethanol (5–30%, vol/vol) were preincubated for 1 hr at 37°C in the absence of substrate. Full activity was determined at each pH value. The activity of the enzyme without preincubation was defined as 100%. Activity was determined at their optimal pH and temperature. Data are expressed as the means of three experiments and the standard deviation for the mean was recorded to be <2%.

The optimal temperatures and thermostability were determined for the TmBglA wild type and mutants in Fig. 3a and c. All mutants prepared were catalytically active, indicating that the selected amino acid residues were not influence for active expression of wild-type TmBglA, but resulted in sight shifts in optimum temperature and heat stability. All enzymes were optimally active at 90°C except for G224A and Y295F of which the optimal temperature was 95°C. Nonetheless, the relative activity of all mutants was higher than that of wild-type TmBglA within the temperature range from 35°C to 95°C except N223C. All mutants were stable up to 85°C that were the same to that of wild-type TmBglA, F414S was even more stable within the temperature range from 70°C to 90°C compared with other mutants, but Y295F exhibited a half-life at 95°C, which is accompanied by the higher optimal temperature (95°C) than TmBglA, indicating that these mutation caused structural perturbation to change its thermal properties of wild-type enzyme.

The pH properties of six mutants and TmBglA were shown in Fig. 3b and d. The pH optima for all mutants were observed at pH 6.2 that are not altered from the pH optima profile characteristic of TmBglA except N223Q. In contrast, N222F, N223Q, N223C, and G224A were found to a decrease in stability within the pH below 5.8 compared to that of TmBglA, whereas stability of Y295F within the pH range from pH 4.2 to 9.0, that of N222F at pH from 6.2 to 9.0, and that of F414S at pH from 4.2 to 6.2, were even more stable than that of wild-type TmBglA.

The kinetic properties of TmBglA and 6 mutants for the hydrolysis of *p*NPG were determined at optimum condition. The results are shown in Table 3. The *K*_m_ (mM) values of N222F and G224A calculated were 0.20 and 0.35, respectively, which were slight lower than that for the TmBglA (0.38), whereas the *K*_m_ (mM) for the N223Q, N223C, Y295F, and F414S were 0.56, 0.40, 0.61, and 2.29, respectively, which were higher than that for the TmBglA. The relatively more pronounced effect on the *K*_m_ however raised an interest for trying these variants in transglycosylation reactions of *p*NPG. N222F, N223Q, N223C, G224A were significantly superior to the TmBglA in catalytic efficiency, and showed an increased turnover *k*_cat_, and catalytic efficiency *k*_cat_/*K*_m_ for *p*NPG; of these, N222F exhibited the highest *k*_cat_/*K*_m_ value for *p*NPG duo a minimum in the *K*_m_ value compared to those of other mutants, and shows the most efficient *p*NPG hydrolysis, which suggests these results correlate well with as observed in previous study on that *T*. *neapolitana* β-glucosidase TnBglA variant N220F significantly increased *k*_cat_ and *k*_cat_/*K*_m_ using *p*NPG hydrolysis (Lundemo et al., 2013). However, the *k*_cat_ and *k*_cat_/*K*_m_ value of Y295F and F414S were much lower than those of wild-type TmBglA, Y295F and F414S were hydrolytically crippled with *p*NPG as substrate with an 85 and 70-fold decrease in apparent *k*_cat_, respectively. Similarly, hydrolysis was severely compromised in the *T*. *thermophiles* β-glycosidase variant Y284F and F401S (Tran et al., 2010), and *Halothermothrix orenii* β-glucosidase variant Y296F and F417S (Hassan et al., 2016), *Agrobacterium* β-glycosidase variant Y298F (Gebler et al., 1995), *T*. *neapolitana* β-Glucosidase variant F414S (Lundemo et al., 2013), and the tyrosine and phenylalanine residue were assigned an important role to fine-tune the position of the nucleophile and to stabilize its transition state during the hydrolysis reaction (Gebler et al., 1995; Tran et al., 2010).

**Table 3.** Kinetic Parameters of the Wild-Type and Mutant Enzymes

Enzyme	*K*_m_ (mM)	*V*_max_ (U/mg)	*k*_cat_ (s^−1^)	*k*_cat_/*K*_m_ (M^−1^ s^−1^)
TmBglA	0.38 ± 0.02	526.42 ± 9.42	452.27 ± 8.25	1.21 ± 0.14 × 10^6^
N222F	0.20 ± 0.01	713.54 ± 13.57	613.43 ± 10.39	3.00 ± 0.50 × 10^6^
N223C	0.40 ± 0.01	753.43 ± 12.03	647.16 ± 9.74	1.61 ± 0.06 × 10^6^
N223Q	0.56 ± 0.04	895.67 ± 14.23	769.72 ± 12.14	1.40 ± 0.24 × 10^6^
G224A	0.35 ± 0.03	718.34 ± 8.58	617.32 ± 6.75	1.77 ± 0.06 × 10^6^
Y295F	0.61 ± 0.09	6.16 ± 0.23	5.29 ± 0.14	8.74 ± 0.51 × 10^3^
F414S	2.29 ± 0.01	7.49 ± 0.01	6.42 ± 0.01	2.81 ± 0.01 × 10^3^

*Note.* The kinetic parameters were determined at their optimal pH and temperature for the substrate concentrations ranging from 0.2 to 2.0 mM for *p*NPG using the standard assay as described in “Materials and Methods.”

The effect of ethanol on TmBglA and mutants activity were investigated (Fig. 3e), three mutants retained above 100% of activity at an ethanol concentration from 5% to 10%, and N222F, N223Q, and Y295F displayed about 139%, 111%, and 104% of activity, N222F and Y295F displayed about 121% and 103% of activity at 10% ethanol, respectively, exhibited much more increase in activity compared with other mutants and wild-type TmBglA. Of these, the effects of ethanol tested on F414S exhibited a most significant decrease in activity, just as the most catalytically impaired F414S with respect to the hydrolytic reaction. In the presence of 15% (vol/vol) ethanol, N222F, N223Q, G224A, and Y295F retained 92%, 66%, 73%, and 96% of the original activity, which were 1.6-, 1.1-, 1.4-, and 1.7-fold of TmBglA, respectively. Y295F retained 90% (20% ethanol) and 68% (25% ethanol) of the original activity when the concentration from 20% to 25% (vol/vol) ethanol, which were 1.7- and 2.0-fold of TmBglA, respectively, exhibited higher ethanol tolerance than TmBglA and others variants. Which we report herein was in accord with that the corresponding variant F297Y from *Gongronella* sp. W5 retained 33% of the original activity in the presence of 15% (vol/vol) ethanol (Fang et al., 2016). The enzymatic synthesis of alkyl-β-glycosides was performed in water–alcohols two-phase system. When using alcohol substrates as acceptor, Y295F and N222F with higher ethanol tolerance are preferred candidates for industrial chemo-enzymatic synthesis: it allows the application of such enzymes in a wider range of solvent environments and by reducing water activity increases the enzymatic synthesis yield of alkyl-β-glycosides via transglycosylation (Seraphim, 2001).

**Fig. 4:**
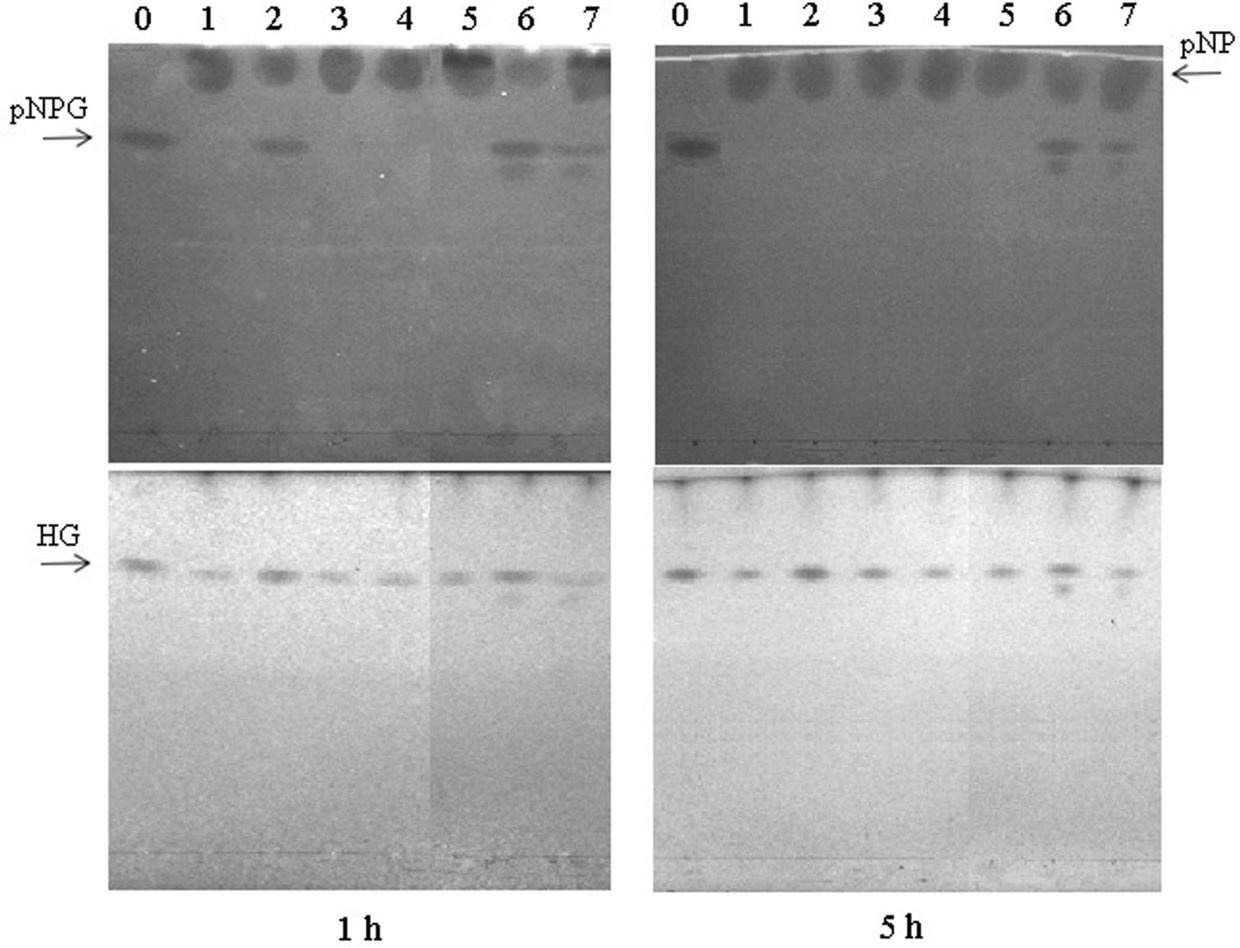
TLC analysis of the transglycosylation products obtained by transglycosylation reaction with the wild-type and mutant TmBglA. The enzymatic reaction was carried out with *p*-nitrophenol β-d-glucoside and hexanol at 60°C for 1 hr (left) and 5 hr (right). Lane 0, without enzyme; lane 1, TmBglA; lane 2, N222F; lane 3, N223C; lane 4, N223Q; lane 5, G224A; lane 6, Y295F; lane 7, F414S.

The transgalactosylation activity of the TmBglA variants was subsequently investigated in more detail using purified enzyme. The reactions enzymes were performed at 60°C with an initial *p*NPGlc concentration of 34 mM using the same amount of purified enzyme. As indicated in Fig. 4, the variant N222F, N223C, N223Q, G224A displays product patterns similar to those of the wild type, whereas Y295F and F414S show different patterns of HGs formed. Particularly, Y295F and F414S mutation increased self-condensation, the yielding *p*-nitrophenol-glucobiose using *p*NPG as a glycosyl acceptor were generated in the reactions for 1 and 5 hr of incubation (Fig. 4). The hydrolysis of *p*NPGlc by TmBglA, N223C, N223Q, G224A were complete after 1 hr of incubation, and exhibited that N223C, N223Q, G224A produced the same HG as the wild type, but the reactions with other mutants N222F, Y295F, and F414S were retained some *p*NPGlc; the accompanying HG product for N222F, Y295F and F414S were higher than those for TmBglA and other mutant. These results are consistent with the slight improvement of transglycosylation activity observed with the *T. neapolitana*, *H*. *Orenii*, and *T*. *thermophiles* β-glucosidase after mutation of the homologous position N220F, Y296F, and F401S, respectively (Hansson & Adlercreutz, 2001; Hassan et al., 2016; Lundemo et al., 2013). This suggests that these residues, or the homologous positions in GH1 glycosidases, are the best targets to improve the transglycosidase activity of this enzyme family.

**Fig. 5:**
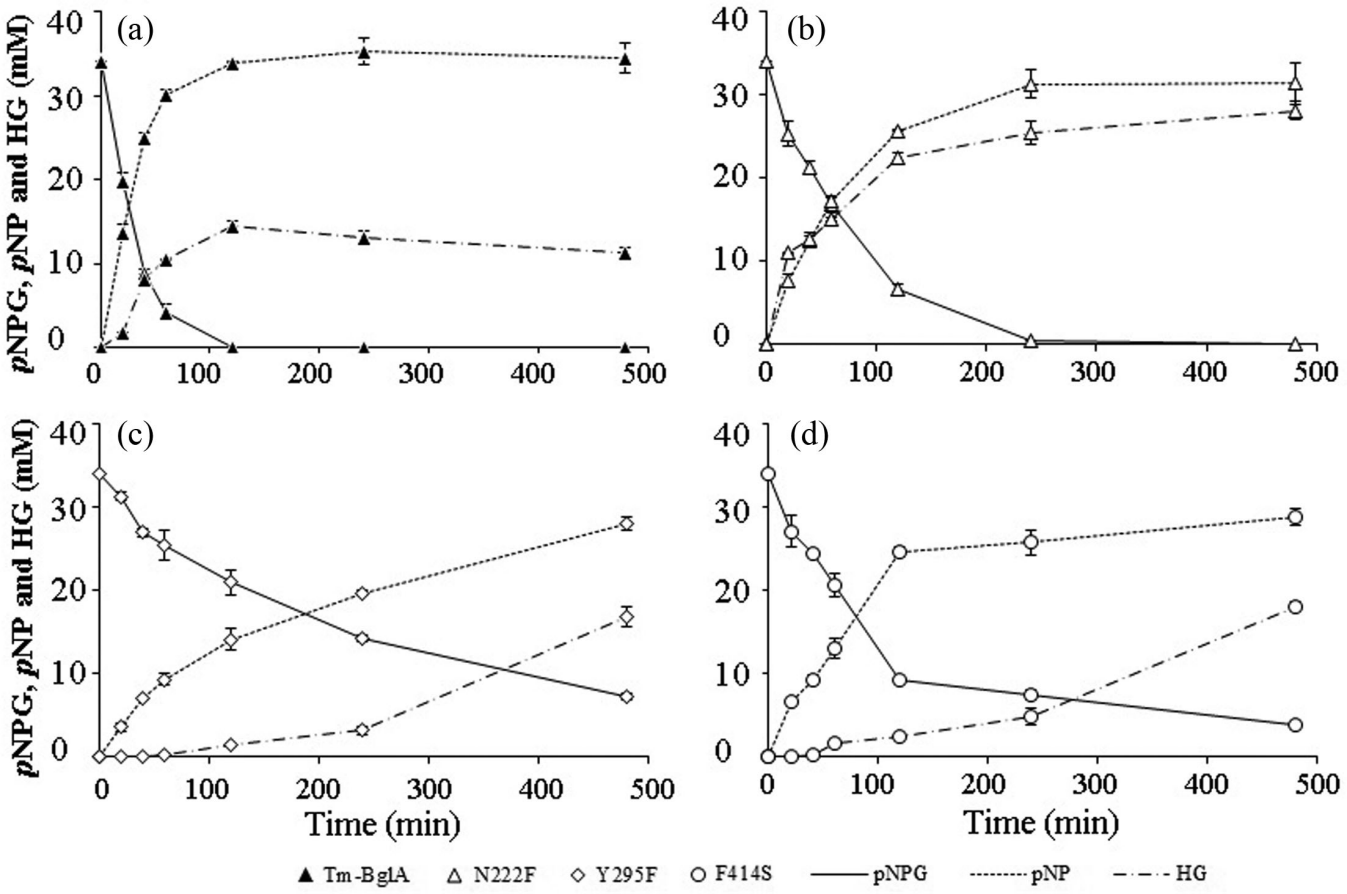
Time course for synthesis of hexyl-glucoside (−∙−) and *p*-nitrophenol (…) from *p*-nitrophenol-d-glucoside (—) catalyzed by TmBglA (▲), N222F (△), Y295F (◊) and F414S (○). Reaction conditions: 34 mM *p*-nitrophenol-d-glucoside and 80% hexanol (vol/vol), 10 μg/ml purified enzyme, 60°C.

On the basis of the aforementioned, the changes of components in the reactions with TmBglA, N222F, Y295F, and F414S using *p*NPG as the glycosyl donor and hexanol as the acceptor were further analyzed by HPLC (Fig. 5). The reactions were conducted at 75°C with an initial *p*NPG concentration of 34 mM using the same amount of enzyme. Under the same condition as depicted in Fig. 3a and b, the transglycosylation product HGs from *p*NPG by N222F, Y295F, and F414S significantly higher than that obtained from TmBglA, and the amounts of HG increased with increasing reaction time. During the initial 20 min of incubation with p-nitrophenol β-d-glucoside *p*NPG, 10.9 mM for N222F and 1.838 mM for TmBglA of hexyl-glucoside (HG) were obtained; it appeared that the conversions from *p*NPGs to HG with N222F were much higher and faster than that with TmBglA, whereas no HG was detected in the reactions with Y295F and F414S. Meanwhile, the *p*-nitrophenol formation rate (hydrolysis) was evaluated, the amount of *p*-nitrophenol for N222F, Y295F, and F414S in the first 20 min of the reactions decreased rapidly to 7.6, 3.5, and 6.6 mM, which were 0.55-, 0.26-, and 0.48-fold of TmBglA (13.7 mM), respectively, exhibited three mutants lower hydrolysis rate in the presence of hexanol, and N222F had more striking ratio of transglycosylation (HG formation rate) over hydrolysis (*p*PN formation rate) than TmBglA. At the 40 min reaction, the amount of HGs increased rapidly to 12.073 mM for N222F and 8.187 mM for TmBglA, their percent conversion had 35.5% for N222F and 24.08% for TmBglA, exhibited that the conversions from *p*NPG to HG with N222F were higher and faster than that with TmBglA. After 2 hr, all *p*-nitrophenol β-d-glucoside was almost completely converted to their products *p*NP (hydrolysis) and HG of (transglycosylation) by TmBglA respectively, and reached the highest value of 33.94 mM of *p*NP and 14.49 mM of HG, and the yield of HG obtained from 2 to 8 hr enzymatic hydrolysis of TmBglA decreased 14.49 to 11.35 mM, and showed hydrolysis of formed HG started slowly, demonstrating that TmBglA had a low hydrolytic activity on HG affecting the yield of HGs. In contrast, N222F produced 25.6 mM of *p*NP (hydrolysis) and 22.8 mM of HGs in 2 hr of incubation, and the HG obtained from 2 to 8 hr enzymatic hydrolysis of N222F increased from 22.8 to 28.806 mM, and the *p*NPGs were almost completely digested, suggesting that N222F mutation eliminated the hydrolytic activity of formed HG to improve HG yield. However, Y295F and F414S produced a very low hydrolytic product (*p*NP), the initial HGs produced was obtained at 60 min for Y295F (0.221 mM) and at 40 min for F414S (0.209 mM), and the yield of HG obtained from 2 to 8 hr enzymatic hydrolysis increased from 1.25 to 17.306 mM for Y295F, and from 2.4 to 18.4 mM for F414S, but some *p*NPGs were still remained in 8 hr of incubation, exhibited that the conversions from *p*NPG to HG with Y295F and F414S were higher and slower than that with TmBglA; N222F, F295Y, and F414S synthesized HG yields of 84.7%, 50.9%, and 54.1%, respectively, Of these, N222F is the highest efficient mutant in hydrolyzing *p*NPG and synthesizing HG compared with the other mutants and the wild type. N222F shows the highest yields of HG product, which increased from 14.49 (TmBglA) to 22.8 mM (N222F) at 2 hr by 57.42%. This is accompanied by its highest *k*_cat_/*K*_m_ value for *p*NPG hydrolytic reactions studied in an aqueous environment. On the contrary, F295Y and F414S produced lower transglycosylation product HG than N222F because of the decreased hydrolytic activity of *p*NPG, as indicted in **Table** **3**.

**Fig. 6:**
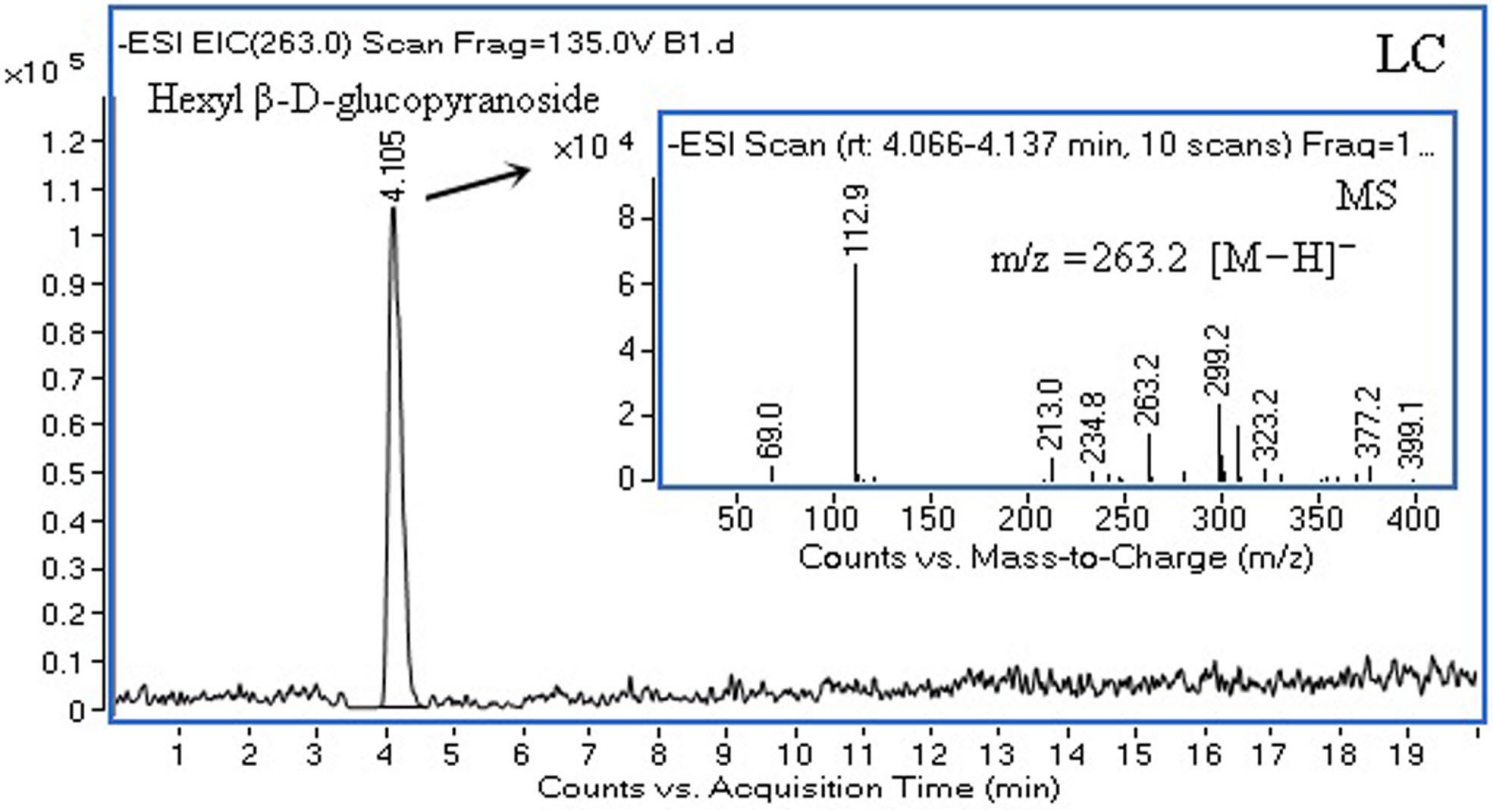
Identification of the transglycosylation products (hexyl-β-glycoside) by using LC–MS.

The end products of enzymatic synthesis using *p*NPG as the glycosyl donor and hexanol as the acceptor after reactions with TmBglA were determined by electrospray LC–MS/MS (Fig. 6). By comparing both retention time and mass spectrum with a standard, a major peak was identified as hexyl β-d-glucopyranoside (molecular ion *m*/*z* 263.1 [M − H]^−^) at 4.050 min of retention time.

### Plausible Explanation of Catalytic Mechanism Based on Molecular Modeling

In order to understand the difference in transglucosylation activity of TmBglA, N222F, Y295F, and F414S, the wild-type enzyme–*p*NPG complex was built and compared with the mutants–*p*NPG complex. According to the relative docking energy criteria (ΔG, −6.41, −7.48, −6.98, and −7.29 kcal/mol for wild type, N222F, Y295F, F414S, respectively), all mutants increase the affinity between enzyme and *p*NPG; as they give a lower free energy value. Of these, the ΔG between the *p*NPG and N222F was least (−7.48 kcal/mol), indicated that *p*NPG–N222F complexes system is most stable, and its substrate affinity is highest, which is consistent with those observed for *K*_m_ in previous study (Sun et al., 2014). In the molecular model, the distances of the N222, Y295, and F414 residues for TmBglA to *p*NPG were 6.58, 9.078, 9.69 Å, respectively; and reduced (−1.1, −1.29, −1.13 Å) by N222F (5.48 Å), Y295F (7.79 Å), and F414S (8.56 Å) mutation to enhance the interaction between the enzyme and *p*NPG, and have less steric hindrance, giving the substrate greater activity space, and resulting in a good conformation. For the two lower *p*NPG hydrolysis mutants (Y295F: 8.74 ± 0.51 × 10^3^ F414S: 2.81 ± 0.01 × 10^3^ M^−1^ s^−1^), this increase in the affinity for *p*NPG promoted that *p*NPG could be used as a glycosyl acceptor, yielding *p*-nitrophenol-glucobiose, an unwanted but common side reaction product during transglycosylation (Feng et al., 2005; Tran et al., 2010), which is verified by our above results of TLC. This may explain why the HG yields of Y295F and F414S is lower than that of N222F in catalyzing synthesis.

**Fig. 7:**
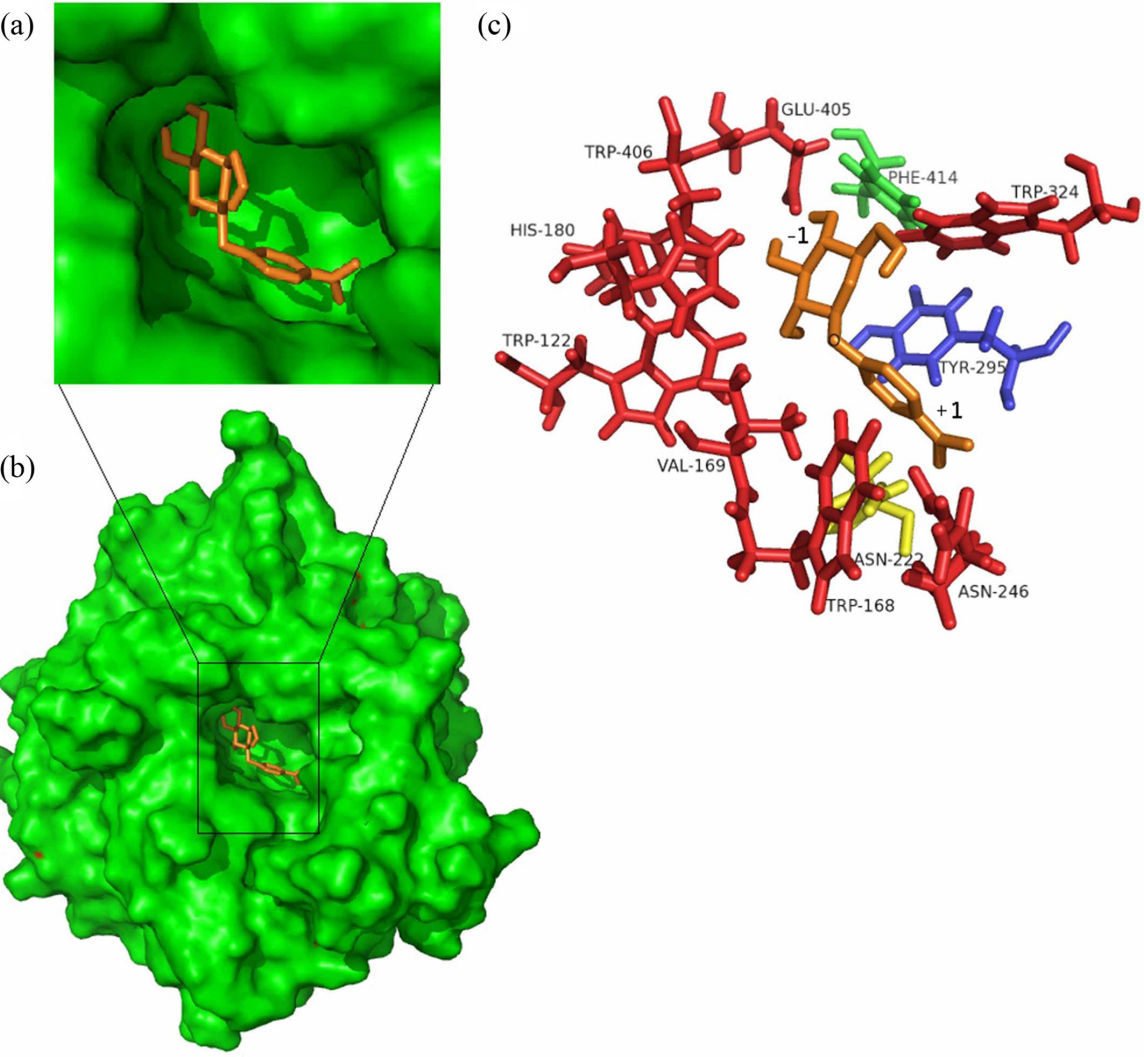
Structural details of mutation sites. Position of N222 (yellow), Y295 (blue), and F414 (green) in TmBglA containing a docked *p*NPG molecule (brown) (color figure online).

Analysis of the three-dimensional structure of TmBglA showed that three efficient mutations, N222F and Y295F are positioned in the aglycone subsite +1, and F414S is located just in the glycone (−1) subsite (Fig. 7). Replacement of N222 and Y295 with the phenol hydroxyl into the +1 subsites is likely to increase the hydrophobicity of the aglycone subsite, favoring hexanol over water and thereby increasing their transferase activity. This agrees with a study that reported an increase its value for synthesis of alkyl glycosides by the phenylalanine substitution at position N220 of a GH1 β-glucosidase from *T*. *neapolitana* in which N220 influences not only glycosyl donor binding but also glycosyl acceptor specificity(Lundemo et al., 2013, 2017). F414S increased transglycosylation activity, likely because this residues is located just in front of the glycone (−1) subsite, which introduced the polar residue serine into the −1 subsite together with a better fit of the acceptor in the (+1) subsite to favor the attack of a glycosyl acceptor in the mutant at the expense of water, which was demonstrated to be responsible for strong enhancement of the transferase/hydrolase ratio by corresponding residue in *T*. *thermophiles* β-glucosidase variant F401S (Feng et al., 2005).

Subsequently, the TmBglA–HG complex was built and compared with the mutants–HG complex to understand the effect of transglycosylation product hydrolysis on transglycosylation activity. According to the relative docking energy criteria, the free energy differences are sufficiently significant to allow interpretation of their difference in transglycosylation activity. For the three higher production mutants (N222F: 28.8 mM, Y295F: 17.3 mM, F414S: 18.4 mM), the ΔG for N222F, Y295F, and F414S were −5.38, −7.29, −6.58 kcal/mol, respectively, and higher than that for the TmBglA (−8.18 kcal/mol), suggesting that all mutation decrease the affinity between enzyme and HG as they give a higher free energy value. Three mutants creates an environment more suited for hexanol in the active site pocket, and consequently suppressed its hydrolysis of the transglycosylation produced HG. Of these, the ΔG between the HG and N222F was the highest, indicated that N222F mutation constitutes the weakest substrate bonding for HG, and resulting in most suppressed its hydrolysis of transglucosylation product HG. This may explain why the N222F is the highest yields of HG in catalyzing synthesis. In the structural model of these HG–enzyme interactions in the wild type and the mutants, the distances of the amino acid residues N222, Y295, and F414 for TmBglA to HG were 4.62, 8.84, 9.93 Å, respectively, and extended (+1.35 Å, +6.25 Å) by N222F (5.97 Å) and F414S (16.18 Å) mutation to reduce the interaction between the enzyme and HG, respectively; compared with Y295F (8.08 Å) mutation with a larger increase (−7.29 kcal/mol) in the affinity between the enzyme and HG, and have greater steric hindrance, giving the HG less activity space, and resulting in a worst conformation and consequently suppressed its hydrolysis of the transglycosylation produced HG. In this study, the yield of HG obtained by N222F was superior to those obtained by TmBglA, Y295F, and F414S, suggested that that transglycosylases tend to increase the hydrophobicity of the aglycone subsite, favoring hexanol over water and thereby increasing the transferase/hydrolase ratio. Similarly, the corresponding TnBgl1A variant N220F improve transglycosylation activity and alkyl glycosides yield (Lundemo et al., 2013). Different β-glucosidases have different synthetic abilities of alkyl glycosides depend on their aromatic residues at a position corresponding to the + 1 subsite of enzyme and the specificity for *p*NPG or HG. Higher ratios of transglycosylation have also been seen in other mutational studies with improved affinity of the hexanol as acceptor in aglycone subsites (Hansson & Adlercreutz, 2001; Hassan et al., 2016; Lundemo et al., 2017; Teze et al., 2014; Tran et al., 2010).

## Conclusions

In conclusion, the residues (W168, N222, N246, Y295, and F414) at the glycone (−1) subsites of β-glucosidases from *T*. *maritima* (TmBglA), were selected for mutagenesis to investigate the influence on the alkyl glycosides production for TmBglA. Our results suggest that the three amino acid residues (N222, Y295, and F414) may modify the interactions in the active site pocket, leading an environment more suited for hexanol as well as conformational changes that allow increased transglycosylation activity and synthesizing HG product using *p*NPG as glycosyl donor and hexanol as acceptors, which have valuable biosynthesis of alkyl glucoside.

## Funding

This work was supported by grants from “National Key Research and Development Project” of China (Grant No. 2019YFA0706900).

## Conflict of Interest

The authors declare no conflict of interest.

## Ethical Approval

This article does not contain any studies with human participants or animals performed by any of the authors.
